# Tumor in an Adult Male Located in the Lower Extremity: A Case Report

**DOI:** 10.1002/ccr3.70491

**Published:** 2025-05-07

**Authors:** Mariana Al Hawa, Serena Saade, William Abou Shahla, Dana Saadeh

**Affiliations:** ^1^ Department of Dermatology American University of Beirut Medical Center Beirut Lebanon

**Keywords:** lower extremity, Masson tumor, pathology, rare tumor, skin disease

## Abstract

Masson's tumor is a rare condition that can mimic malignancy, particularly when located in atypical regions. This case emphasizes the importance of considering this diagnosis in unusual anatomical locations and highlights the critical role of histopathological evaluation in achieving an accurate diagnosis. Prompt identification and surgical management can lead to favorable outcomes, even in rare presentations.

## 1 Introduction

Masson's tumor, also known as intravascular papillary endothelial hyperplasia (IPEH), is a benign non‐neoplastic lesion, first described in 1923 under the name of “vegetant intravascular hemangioendothelioma” [1]. Initially believed to be an atypical proliferation of papillary endothelial cells, it was reclassified as a reactive process in 1932 by Henschen. This tumor is rare and accounts for 2% of benign and malignant vascular tumors of the skin and subcutaneous tissues [2].

This case highlights a Masson tumor, a vascular tumor arising in the lower extremities, a region where such tumors are less commonly observed compared to their typical occurrence in the head and neck area.

## 2 Case History/Examination

A 49‐year‐old male hairdresser presented to an outpatient clinic in Beirut, Lebanon, with a chief complaint of a lesion on the right posterior lower extremity, which had been present for the past 3 years. The lesion had grown recently, over the last 6 months, and was not painful to touch. The patient's occupation required prolonged periods of standing.

**FIGURE 1:**
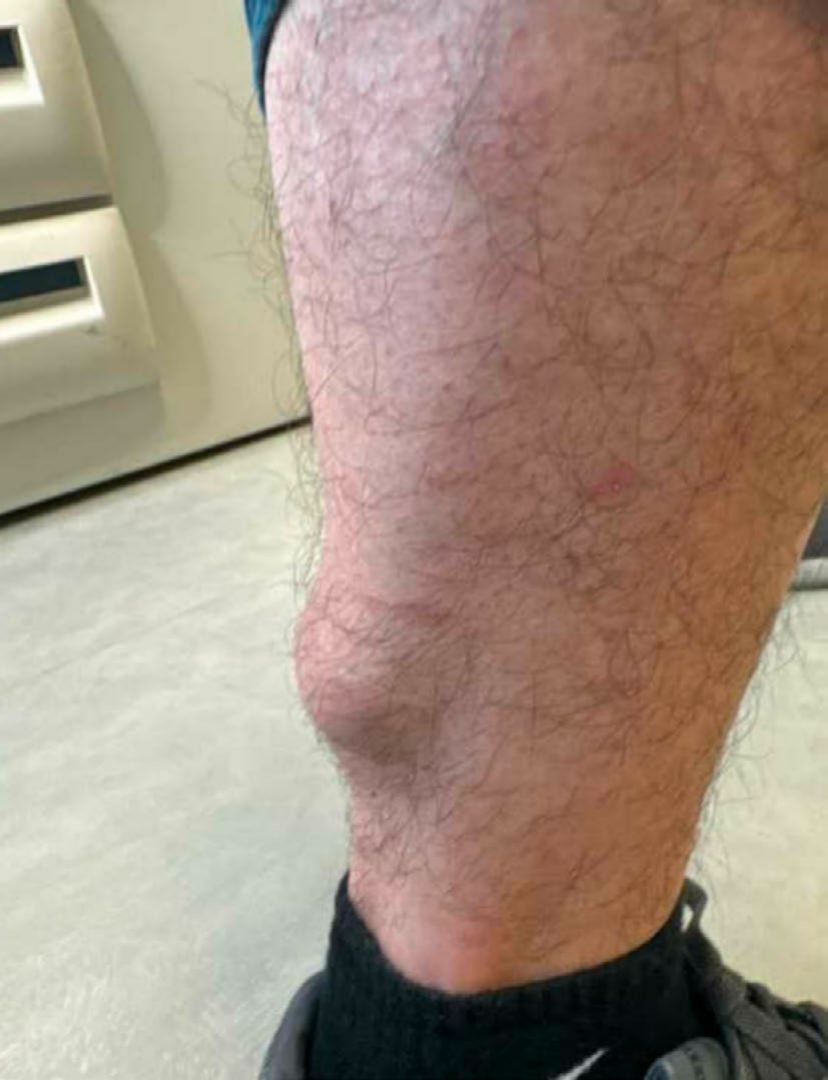
Photograph of the well demarcated lesion on the posterior calf of the right extremity of the male patient in his 40s.

On examination, a large, soft, well‐demarcated subcutaneous nodule with a benign clinical appearance, suggestive of a common soft tissue lesion such as a lipoma, measuring approximately 4 × 4 cm in size was noted at the site (Figure 1). The lesion was non‐tender to palpation. No additional symptoms or concerning features, such as rapid growth, pain, or skin changes, were observed. The patient had no significant past medical history, surgical history, or reported allergies. Family history included pilar cysts in the patient's mother and brother.

## 3 Methods

### 3.1 Differential Diagnosis

Based on the clinical presentation, the differential diagnosis primarily included lipoma, epidermoid cyst, or a benign vascular lesion.

### 3.2 Investigations

Due to financial limitations, advanced imaging or biopsy was not performed prior to excision. The clinical evaluation supported the decision to proceed with direct surgical excision under the working assumption of a lipoma.

### 3.3 Treatment

The nodule was excised in its entirety during the initial surgical procedure. Complete surgical excision was achieved, as confirmed by histopathology, with no evidence of residual lesion or malignancy.

Post‐excision, the gross examination revealed a well‐demarcated vascular nodule, located superficial to the subcutaneous tissue, comprising three lobules encapsulated within fibrous bands. Histologic examination of the excised tissue revealed a dilated vessel within the superficial subcutaneous tissue, showing intravascular proliferation of reactive endothelial cells. The endothelial cells formed numerous papillary structures lined by a single layer of plump endothelial cells. The lesion was enclosed within a fibrous pseudo capsule, supporting the diagnosis of a benign vascular tumor.

## 4 Conclusion and Results

**FIGURE 2:**
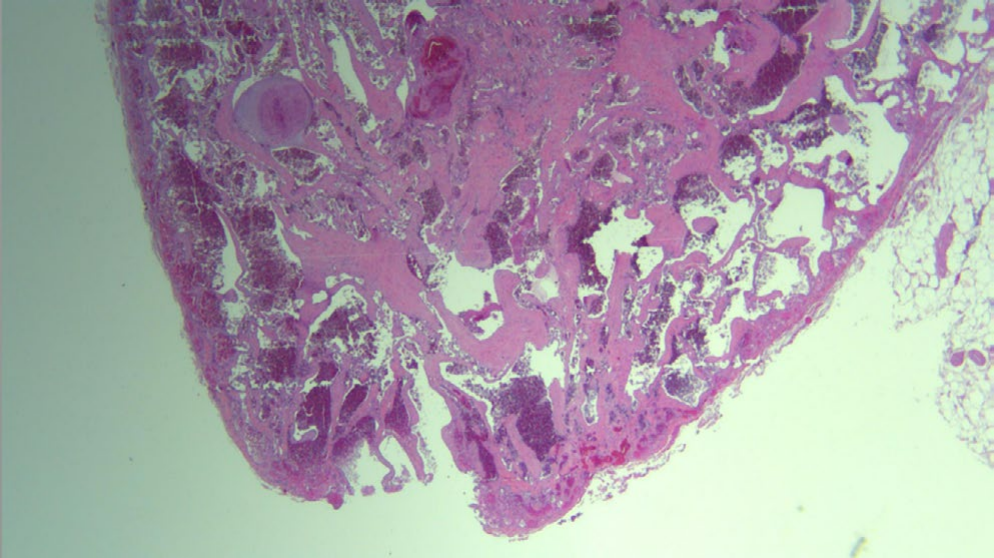
Histopathological slide of the tumor.

**FIGURE 3:**
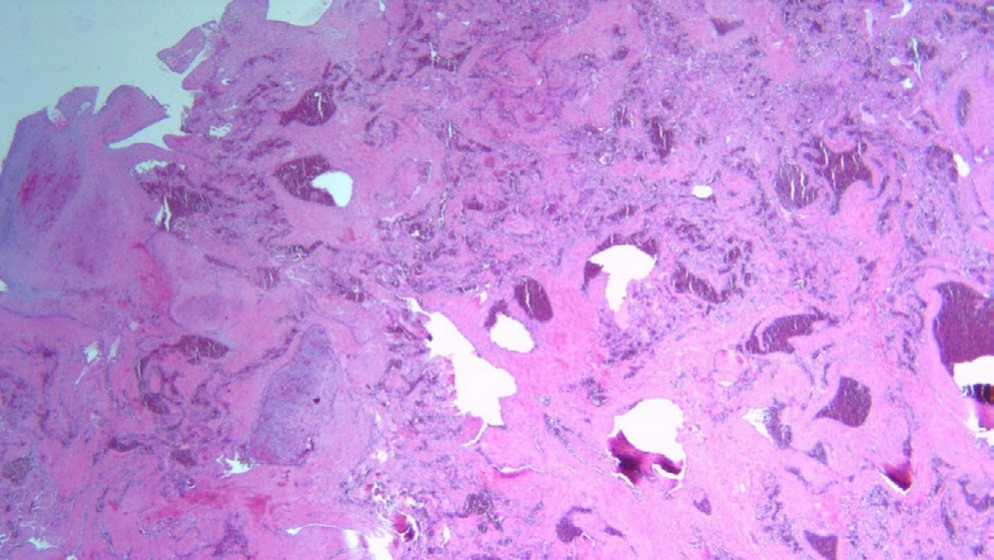
Histopathological slide of the tumor with a higher magnification showing the papillary structures, located within vascular spaces, are lined by a single layer of plump endothelial cells, with fibrous connective tissue cores.

Figures 2 and 3 show the histological results. The surgical approach and prognosis were explained to the patient.

Following complete surgical excision, the patient experienced an uneventful recovery with no signs of recurrence at follow‐up. Dermatologists and surgeons should maintain a high index of suspicion for IPEH when evaluating vascular tumors to optimize patient outcomes.

Intravascular papillary endothelial hyperplasia (IPEH) is a rare but benign vascular lesion that poses diagnostic challenges, particularly in the clinical and preoperative settings. Accurate diagnosis relies heavily on histopathological evaluation, as clinical presentations often mimic other soft tissue lesions. This case underscores the importance of considering IPEH in the differential diagnosis of vascular tumors to ensure timely and appropriate management.

## 5 Discussion

This tumor has several names, including Masson's hemangioma, Masson intravascular hemangio‐endothelioma, intravascular papillary endothelial hyperplasia (IPEH), and reactive papillary endothelial hyperplasia [3].

The precise etiopathogenesis remains unclear, though it is widely regarded as a reactive process believed to result from growth factors production, leading to excessive endothelial cell proliferation [4]. In fact, research employing northern blot and immunoblotting revealed a significant increase in the expression of fibroblast growth factor beta, suggesting its secretion by the endothelial cells through an autocrine mechanism. The condition is thought to arise from an aberrant resolution of a thrombosis, unfolding through several stages [5].

It typically manifests as a firm or soft, reddish‐blue color nodule over the head and neck and upper extremities [6]. Occurrences on the lower extremities, such as in our case, are rare. Generally, it is asymptomatic, slow‐growing, and is not associated with any pulsation. The sonographic features of subcutaneous IPEH are not specific; however, consideration of the diagnosis is warranted when encountering a soft tissue mass showing a vascular pattern. This entity has a slight predilection for adult females, and IPEH can also manifest in children [7]. An interesting case published in 2009, of a 1‐month history of IPEH in the calf of a 1‐year‐old infant, highlights a mixed form IPEH, which is uncommon in infancy, where MRI showed a recurrence after 6 months of surgical excision. MRI findings of IPEH typically show intermediate signal intensity on T1‐W images and heterogeneous high intensity on T2‐W images, with nodular low‐intensity areas corresponding to chronic thrombi. These imaging characteristics help differentiate IPEH from other neoplasms. Histopathological evaluation is crucial to distinguish IPEH from angiosarcoma, as treatment strategies differ significantly. To minimize the risk of recurrence, a wide excision including surrounding muscle is recommended [8, 9].

Diagnosis mainly relies on histology since imaging such as MRI or US cannot differentiate it from other vascular lesions. Histologically, it is characterized by the presence of papillary structures lined by a single layer of endothelial cells. There will be an absence of pleomorphism, mitosis, or necrosis.

Masson tumor has been classified into three main types. The first type is the pure form, where the endothelial proliferation occurs from veins and less commonly from arteries without any vascular abnormalities. The second type arises from a pre‐existing vascular abnormalities such as an aneurysm [5], arteriovenous malformations, hemangiomas, and pyogenic granulomatosis. The third and least common type arises from an extravascular hematoma. The pure form of intravascular papillary endothelial hyperplasia (IPEH) most commonly affects the fingers, followed by the head, neck, and elbow to hand, and is confined to the subcutaneous tissue. The mixed form, found in approximately half of cases, can occur intramuscularly without a specific site preference. IPEH typically affects patients around 34 years of age, with mixed forms being more common in younger individuals [9].

These tumors stain positive for CD31 and CD34 [10]. Staining positivity for factor VIII, type 4 collagen, and SMA and MSA varies and is not as consistent.

Clinically, IPEH can mimic a variety of conditions, both benign and malignant, such as pyogenic granuloma and Kaposi's sarcoma. Moreover, it should be distinguished from malignant angiosarcoma, a differentiation typically achieved through histology and immunohistochemical staining. The primary characteristic of IPEH is its intravascular development, in contrast to angiosarcoma, which rarely remains confined within the vascular lumen. Also, CD105 is a useful immunohistochemical stain, as angiosarcoma would stain positive while IPEH does not [10].

Since IPEH can be mistaken for angiosarcoma and other malignant tumors, surgical excision is the treatment of choice. Since these tumors rarely occur de novo within extravascular hematomas, meticulous surgical techniques should be utilized during resection to prevent any spillage of the aneurysm‐associated thrombus, given the theoretical risk of extravascular recurrence. Other alternative options include observation or using sclerosing agents such as sodium tetradecyl sulfate [11]. Chemotherapy or radiotherapy may be considered for cases of recurrence following incomplete excision, as well as for multiple intracranial lesions or suspected lesions [12].

## Author Contributions

**Mariana Al Hawa:** methodology, project administration, resources, writing – original draft, writing – review and editing. **Serena Saade:** writing – original draft, writing – review and editing. **William Abou Shahla:** methodology, resources, writing – original draft, writing – review and editing. **Dana Saadeh:** writing – review and editing.

## Ethics Statement

The patient in this manuscript has given written informed consent for participation in the study and the use of their de‐identified, anonymized, aggregated data and their case details (including photographs) for publication.

## Consent

The authors obtained written consent from patients for their photographs and medical information to be published in print and online and with the understanding that this information may be publicly available. Patient consent forms were not provided to the journal but are retained by the authors.

## Conflicts of Interest

The authors declare no conflicts of interest.

## Data Availability

The data that support the findings of this study are available upon request from the corresponding author. The data are not publicly available due to privacy or ethical restrictions.
